# Anesthesia, perimortem conditions, and brain harvest method govern the measured metabolome: analysis of substandard samples generates flawed metabolite levels

**DOI:** 10.1093/oons/kvag011

**Published:** 2026-08-26

**Authors:** Gerald A Dienel

**Affiliations:** Department of Neurology, University of Arkansas for Medical Sciences, 4301 W. Markham Street, Little Rock, AR 72205, United States

**Keywords:** Alzheimer’s disease, brain harvest, [^13^c]glucose metabolite profiling, metabolome, neuronal glycolytic upregulation, Postmortem ischemia

## Abstract

Metabolism and energetics are closely integrated with brain function, and understanding how metabolic dysfunction contributes to neurological disease may help improve therapeutic approaches. Characterization of *in situ* metabolite levels, spatial distributions, and metabolomes requires special attention to anesthesia use, perimortem conditions, and tissue harvest. Anesthesia, CO_2_ asphyxiation, vascular perfusion, and decapitation alter metabolism, thereby substantially changing the metabolome, lipidome, and neurotransmitter profile. Analysis of flawed samples produces artifactual data, raising concerns about conclusions regarding the cellular basis of metabolism, metabolite shuttling, pathway activities, labeled metabolite profiling and imaging, and relationships between metabolism, function, and neurological disorders. Appropriate procedures for in vivo metabolic assays and tissue harvest are widely-unrecognized but essential components of metabolic and metabolomic studies.

## Introduction

Brain metabolism and energetics are integrated with neural functions at a local level (Dienel 2019a), and a detailed understanding of metabolomic alterations in neurological diseases may help improve therapeutic approaches. Accurate characterization of the metabolic profile, metabolome, and spatial images of metabolite levels to define metabolic dysfunction requires that metabolites in living tissue are properly measured and tissue samples are appropriately harvested. This concern applies to all tissues, not just to brain that is emphasized in this commentary. The impact of anesthesia on metabolism and function are well known, and stopping metabolism at tissue harvest is critically-important issue that is widely unappreciated in spite of its being common knowledge in the brain metabolism field for almost 100 years. In fact, ~95% of 780 metabolomics publications that specified a harvest procedure used an inappropriate method (see supplemental file 1 in reference Dienel and Nowak Jr 2025). If the starting sample is not valid, the results of its analysis with advanced analytical and imaging methodologies are flawed, and the knowledge base is not advanced.

## Anesthesia alters metabolic rates and metabolite levels

Many studies have documented the complex actions of anesthetics on neurotransmission, neuronal activity, and the functional or cognitive status of the anesthetized subject (reviewed by Müller et al. 2011, Masamoto and Kanno 2012). Metabolic rates in anesthetized or immature subjects are lower, and postmortem changes are slower than in awake adults (Lowry et al. 1964, Gatfield et al. 1966, Goldberg et al. 1966, Kauffman et al. 1969). Anesthetics that produce graded levels of functional depression based on changes in EEG activity also proportionately reduce brain glucose oxidative metabolism and excitatory neurotransmission (Sibson et al. 1998). The metabolic profiles as components of the total metabolome characterized in living brain by proton (^1^H) magnetic resonance spectroscopy (MRS) under isoflurane or propofol anesthesia are different (Makaryus et al. 2011), and the metabolomes in extracts of murine heart, skeletal muscle, liver, adipose tissue, and serum are differentially affected by anesthesia (Overmyer et al. 2015). Thus, use of anesthesia during metabolic assays or just prior to or during euthanasia increases the complexity of comparisons between control and experimental groups of metabolic profiles in living brain and of the overall metabolomes determined in tissue extracts.

Anesthesia use adds a requirement for investigators, i.e. determine whether group differences report actual *in situ* metabolite levels in awake subjects or arise from different responses to the anesthetic. In other words, additional controls are required to establish the effects of anesthesia in each experimental paradigm. For example, Dalsgaard et al (Dalsgaard et al. 2007). measured glycogen in biopsy samples of hippocampus from patients with epilepsy who underwent curative surgery. As controls, hippocampal biopsy samples from normal anesthetized pigs and tissue from guillotine freeze-clamped tissue from awake and anesthetized rats were analyzed in parallel with the human samples (Dalsgaard et al. 2007), while noting that different anesthetics were used in the animals.

## Appropriate tissue harvest protects labile metabolites

### Perimortem conditions

Stressful handling, restraint, sensory stimulation due to laboratory conditions and noises, and other situations can influence metabolite levels and must be described in detail. Use of anesthesia prior to euthanasia or during perfusion of the brain to remove blood will influence the metabolome. Perfusion of the brain followed by decapitation and other tissue processing (e.g Belanger et al. 2018, Gupta et al. 2022, Loppi et al. 2023) causes perimortem (no oxygen or glucose in the perfusate) and postmortem (no blood flow) ischemia, and perfusion may wash out diffusible metabolites. As discussed below, CO_2_ asphyxiation disrupts metabolism prior to death and would alter postmortem ischemic changes in the metabolome.

### Postmortem ischemia

Blood flow stops at death, preventing delivery of oxygen and glucose to brain cells and removal of metabolic waste products, i.e. ischemia. Lack of fuel initiates cascades of metabolic changes in many pathways, quickly resulting in energy failure. Any lag between death and stopping enzymatic activity causes postmortem ischemic changes. The longer the postmortem interval, the greater the concentration changes and pathway involvement.

### Enzyme inactivation

#### Brain

Enzymatic activity must be stopped by rapid freezing or heating at, *not after*, tissue harvest. Early studies used decapitation into Freon 12 cooled to -150°C, with vigorous stirring; this is a passable, but sub-optimal (e.g. some phosphocreatine (PCr) is lost) procedure for small rodents (Lowry et al. 1964, Goldberg et al. 1966, Ferrendelli et al. 1972). Brain harvest methods were since improved: *in situ* or funnel-freezing (Pontén et al. 1973a, Pontén et al. 1973b), guillotine freeze-clamping (Bolwig and Quistorff 1973, Quistorff 1975), freeze-blowing (Veech et al. 1973), and focused microwave fixation *in situ* that heats tissue to 85–95°C within ~1 s (Medina et al. 1975, Wasek et al. 2018, Juras et al. 2023). Many studies report brain harvest followed by ‘flash’ freezing, but ischemic changes have already occurred while enzymes were active.

Each method has advantages and disadvantages, and the goals of a study and technical capabilities of the laboratory influence which method is most appropriate. Freeze-blowing and guillotine freeze-clamping rapidly freeze brain of unanesthetized animals, but freeze-blowing destroys brain structure and guillotine freeze-clamping harvests selected segments. Funnel-freezing requires anesthesia, but nitrous oxide or intravenous, rapid-acting thiopental minimize its impact. All freezing methods require special techniques to prevent thawing during processing and extraction (Passonneau and Lowry 1993). Microwave fixation has the advantage that anatomy is preserved, regions are readily dissected, and routine extraction procedures can be used. A key issue is thermal sensitivity of specific metabolites during microwave fixation (see (Golovko and Murphy 2008, Wasek et al. 2018)) or analytical procedures in GC/MS assays (Fang et al. 2015). Importantly, microwave fixation yields lower ATP concentrations compared with freeze-blowing (Srivastava et al. 2012). Nevertheless, microwave fixation is the method of choice due to ease of tissue handling and regional analyses. If values for specific compounds are critical, more than one harvest method should be used.

#### Peripheral tissues and biopsy samples

Variations in freeze-clamping procedures (Wollenberger et al. 1960, Palladino et al. 1980) for collection of tissue from other organs *in situ* or from in vitro perfused tissues have also been used for metabolic studies during the past 65 years, noting that the animals are anesthetized for exposure of tissue prior to freeze clamping, e.g. liver (Rennie et al. 1976, Quistorff and Chance 1980, Quistorff and Poulsen 1980), kidney (Maksimovic et al. 2019), muscle (Rennie et al. 1976, Hespel and Richter 1990), and heart (Podzuweit et al. 1978, Andres et al. 2020).

Needle biopsies can sample muscle under local anesthesia and a brief delay between tissue removal and freezing is unavoidable (e.g (Klausen and Sjøgaard 1980, Helge et al. 2007)). Freezing is also used to quench metabolism in brain biopsy samples, noting that surgical levels of anesthesia are necessary (Kirsch and Leitner 1967, Dalsgaard et al. 2007).

#### Tissue slices and cultured cells

Most attention regarding tissue harvest has focused on stopping enzymatic activity in the intact tissue by freezing or heating, but the same principles and concerns also apply to harvest of tissue slices and cultured cells, issues that are rarely taken into account or evaluated experimentally. These preparations are very useful to evaluate metabolic profiles, mechanisms, and specific functions, but have limitations especially for metabolic studies. For example, brain slices incur deaffrentation injury and ischemia during preparation, their CMR_glc_ is about half that in living brain, they depend on diffusion for delivery of glucose and oxygen from the medium, and slice recovery after preparation involves reactive astrocytic changes, loss of astrocytic processes, and other changes (Takano et al. 2014). Cultured cells are obtained from very immature animals that have not attained adult levels of metabolic enzymes, and they are cultured for short periods (~1–3 weeks) in diabetic levels of glucose. Removal of the slice perfusion fluid or cell culture medium deprives the cells of oxygen and glucose, and triggers the onset of perimortem anoxia-aglycemia that persists until samples are frozen or heat inactivated. Washing the samples to remove the medium is a common procedure that increases ischemic duration and risks washout of metabolites (e.g. glucose, pyruvate, and lactate) via equilibrative transporters unless transporter blockers are included in the wash. A recent innovative procedure for fixation of brain slices utilizes rapid heat inactivation (Miller et al. 2023, York et al. 2024).

### Validation and reporting units

Regardless of harvest method, fixation validity must be verified by reporting absolute concentrations of reference metabolites to ensure that postmortem ischemia has been prevented, such as ATP, ADP, PCr, Cr levels and ATP/ADP and PCr/Cr ratios or levels of other labile metabolites (Dienel and Nowak Jr 2025). Metabolite data are most useful when reported as absolute values that can be compared to the literature. Stating the absolute levels in a metabolome (Sylvestre et al. 2022) allows data assessment, and the low reported ATP/ADP and PCr/Cr ratios (Sylvestre et al. 2022) may be due to microwave irradiation (Srivastava et al. 2012) and/or inadequate fixation and ischemia. Ratio values, fold changes, arbitrary units, peak heights or areas, and relative abundance are much less useful because actual concentrations are not provided, requiring readers to evaluate the material. For example, a brain imaging mass spectrometry study by Kleinridders et al (Kleinridders et al. 2018). reported relative abundances and very low ATP/ADP ratios (range ~0.01–0.6 for gray and white matter). Comparison of these data with literature ratios determined by enzymatic assays of extracts of rat, cat, and mouse brain after immersion freezing, funnel freezing, or freeze blowing (8.6 ± 3.2 (SD), n = 27) revealed postmortem ischemic artifacts due to energy failure and ATP consumption to increase ADP level (Dienel 2020).

Translation of data obtained from human brain-bank tissue to the living brain is especially difficult due to long postmortem intervals (PMI) and extensive changes in levels of many metabolites immediately before and after death. Glucose levels and metabolic data derived from Alzheimer’s disease (AD) patients (PMI range 2–33 h) were reported in a featured article by An et al (An et al. 2018). that was highlighted (Talan 2017) and led to claims that AD patients have elevated brain glucose level and abnormal glucose homeostasis before clinical symptoms arise. However, reporting log[glucose concentration] in Fig. 2 in reference (An et al. 2018) masked the true glucose levels that must have arisen from autolytic changes (Dienel 2019b) because essentially all glucose is consumed within ~30s after death (Lowry et al. 1964). Other studies of metabolite abundance in AD brain (PMI, 4–13 h) by Xu et al (Xu et al. 2016a, Xu et al. 2016b) also reported elevated regional glucose levels, along with many other changes in the AD metabolome, but data interpretation is confounded by reporting as fold-change or log_2_[glucose] (Dienel 2019b). Levels of labile metabolites in postmortem brains of subjects with neurological diseases do not reflect those prior to death, and matching the PMI range with that for healthy subjects does not control for disease-related differences in peri- and postmortem metabolism and autolysis.

## Ischemia causes rapid changes in labile metabolite levels and pathway fluxes

Cervical dislocation, decapitation, and dissection (e.g Huang et al. 2019, Wang et al. 2022, Huang et al. 2024) cause postmortem ischemia in all body tissues, and distort both metabolite levels and isotopic labeling. Labile metabolite concentration changes during postmortem ischemia were reviewed in (Murphy 2010, Dienel 2019b, Dienel 2020, Dienel 2021, Dienel and Nowak Jr 2025) that cite an extensive literature available for interested readers, investigators, and reviewers. Concentration changes after decapitation are fast, and altered glycolytic, pentose phosphate shunt pathway, and TCA cycle metabolite levels were detectable at 4–6 s when glycolytic rate had increased ~7.4-fold, larger changes occurred with increasing time after decapitation (Lowry et al. 1964, Goldberg et al. 1966, Kauffman et al. 1969). Examples of labile metabolites and likely artifacts in studies that used inappropriate harvest methods are briefly described below.

### Glucose, glycolysis, and energy metabolites

Between 1935–1942, Kerr and colleagues demonstrated that glucose, glycogen, lactate, PCr, and ATP were labile compounds, and determination of their concentrations required freezing the brain *in situ* (Avery et al. 1935, Kerr 1935, Kerr and Ghantus 1936, Kerr and Ghantus 1937, Kerr and Seraidarian 1942). Lowry and colleagues later established detailed time courses and magnitudes of postmortem changes of glucose metabolites in the glycolytic (Lowry et al. 1964), pentose phosphate shunt (Kauffman et al. 1969), and TCA cycle (Goldberg et al. 1966) pathways. At ~10 s after decapitation, glucose level fell by ~60%–75% and lactate increased 1.8-fold; glucose was nearly depleted by 30s, followed by PCr, ATP, and glycogen at 60s when lactate was elevated ~4-fold (Lowry et al. 1964, Goldberg et al. 1966).

These findings contradict the statement by Hanrieder et al (Hanrieder et al. 2025) that Ma et al (Ma et al. 2025) showed that rapid brain dissection within 10s was sufficient to preserve metabolite stability. Notably, Ma et al (Ma et al. 2025) cervically dislocated then decapitated the mice. The brains were removed from the skull within 10s, rinsed with PBS and given two washes with deionized water, followed by slow freezing in isopentane over 7 min. These brain samples were subjected to postmortem ischemia, and metabolic stability was not proven.

Lactate is a labile metabolite, and its levels measured in brain extracts of wildtype and AD model mice after perfusion of the brain (Zhang et al. 2018, Lu et al. 2019) are affected by anesthesia during perfusion and by peri- and postmortem ischemia. Absolute values for lactate in wildtype mice reported by Zhang et al (Zhang et al. 2018) were 11.5 × 10^−5^ mmol/g in Fig. 4A and 11.5 mmol/g in the text of reference (Zhang et al. 2018); Fig. 2 of Lu et al., reference (Lu et al. 2019) shows lactate was ~0.6 mmol/g. None of these values are correct. Brain lactate levels range from ~0.6–1.5 μmol/g in funnel-frozen, microwave-fixed, and freeze-blown brain (Veech et al. 1973, Miller et al. 1975, Dienel et al. 2002, Srivastava et al. 2012). Also, there may be some pyruvate/lactate washout from brain to the perfusate that may differ in wildtype versus AD mice that were reported to have lower expression levels of monocarboxylic acid transporter (MCT) isoforms MCT1, MCT2, and MCT4 (Zhang et al. 2018, Lu et al. 2019).

To study glycolysis by labeling brain metabolites in vivo, Jimenez-Blasco et al (Jimenez-Blasco et al. 2024). injected [U-^13^C]glucose intraperitoneally, and measured labeled and unlabeled labile metabolites in brain extracts, e.g. glucose-6-phosphate (Glc6P), fructose-6-P (Fru6P), fructose-1,6-bisphosphate (Fru1,6BP), phosphoenolpyruvate (PEP), pyruvate, and lactate. They did not state the labeling duration, measure the time courses arterial plasma specific activity of glucose to account for precursor levels in the experimental groups, or identify the brain harvest method. Microwave fixation and freezing procedures involve specialized techniques that were not described, so it is reasonable to assume the mice were decapitated, causing ischemia-evoked artifactual changes in labile metabolite levels and metabolomic data in the volcano plot and heat map (Jimenez-Blasco et al. 2024). Of interest, previous studies by Lowry and colleagues (Lowry et al. 1964, Goldberg et al. 1966) revealed that postmortem activation of phosphofructokinase (PFK) causes biphasic increases and decreases in Fru1,6BP content, peaking at ~5–10 s and ~2 min, contrasting the progressive decreases in Glc6P, Fru6P, and citrate, and increases in pyruvate and lactate levels. Thus, the concentrations of these unlabeled and labeled compounds and their ratios, e.g. (Fru1,6BP/Fru6P), (Fru1,6BP/Glc6P), (PEP/Glc6P), (pyruvate/Glc6P), and (lactate/Glc6P), vary moment-to-moment after death. The actual values are dependent on the exact but unknown duration of postmortem ischemia in the Jimenez-Blasco et al (Jimenez-Blasco et al. 2024). study. The reported metabolite levels, metabolite ratios, and related data (i.e., Fig. 1e, Extended Data Figs. 1 g and 2e in reference Jimenez-Blasco et al. 2024), do not correspond to their true *in situ* values nor do they report glycolytic activity with respect to genotype. The increase in the Fru1,6BP/Fru6P ratio does not distinguish between PFK activation in vivo and activation in postmortem ischemic brain, and underscores the importance of comparison of data with the literature.

### Amino acids

The concentrations of most brain amino acids in rat brain are relatively stable for ~2–5 min after death, with the exceptions that alanine and γ-aminobutyric acid (GABA) levels rise, whereas the levels of many amino acids change during the postmortem 10 min-48 h span (Perry et al. 1981). There are, however, regional differences in altered amino acid levels when microwave fixed tissue is compared to decapitation (Miller et al. 1990).

The relative stability of amino acids for a few minutes after decapitation enables studies of their levels in animal models for amino acidurias, but this stability does not apply to labile metabolites. For example, the overall metabolomes of phenylketonuria-model mice are not reliable when determined in extracts of brain harvested by an unidentified method and snap frozen (Lu et al. 2020) or after CO_2_ asphyxiation (Dobrowolski et al. 2022) that alters the perimortem and postmortem concentrations of many compounds (see below for more details).

### Lipid and lipid-signaling molecules

In the early 1970s, Bazán (Bazán Jr 1970, Bazán Jr et al. 1971) showed that free fatty acid levels increased by ~3-fold within 30s after decapitation, whereas triacylglycerol content fell by a smaller magnitude, indicating that triglyceride degradation was not the only source of the free fatty acids. Many studies subsequently documented the labilities of lipids and lipid signaling compounds (Dienel 2021), and microwave fixation is considered the best method for lipid studies (Bazinet et al. 2005, Golovko and Murphy 2008, Murphy 2010, Trépanier et al. 2017).

Perfusion of brain of isoflurane-anesthetized mice followed by regional dissection (Loppi et al. 2023) causes perimortem ischemia, with isoflurane contributing to artifactually altering glucose metabolism (see below), and perfusion ischemia disrupting glucose, fatty acid, and neurotransmitter metabolism, followed by postmortem ischemia that further distorts the overall metabolome.

### Neuromodulators and neurotransmitters

Adenosine and cAMP are very labile, quickly increasing in concentration after death (Guidotti et al. 1974, Winn et al. 1979, Delaney and Geiger 1996). Acetylcholine concentration fell by 50% and choline levels rose 4.5-fold during 2 min ischemia (Jope and Jenden 1979). Levels of neurotransmitters and their metabolites are most reliable in microwave-fixed brain, and are considerably disrupted by CO_2_ asphyxiation (Wasek et al. 2018).

Transmitters and their metabolite concentrations obtained in extracts derived from inappropriate tissue harvest methods (e.g Lu et al. 2020, Dobrowolski et al. 2022, Loppi et al. 2023) do not correspond to those *in situ*.

### Neuropeptides, phosphoproteins, proteins

Substance P, neurokinin A, neurotensin, neuropeptide Y, vasoactive intestinal peptide, phosphoproteins, and various proteins have lower concentrations (range: ~20%–60%) within 1–2 min after death compared with those in microwave-fixed brain (Dienel 2021).

## Commonly-used procedures alter the metabolome and ^13^C-labeling

Metabolomic studies that use inappropriate assay procedures or harvest methods may implicitly infer that (i) measured data are equivalent to those *in situ* in the awake state, and (ii) control and experimental groups respond identically to anesthesia or harvest procedures. Neither assumption is correct. The data actually report and compare the effects of anesthesia, perimortem disruption of metabolism, perimortem ischemia, and/or postmortem ischemia in the control and experimental groups. Neurological diseases involve metabolic dysfunction, and responses of diseased tissue to anesthesia and ischemia should differ from those of normal tissue. Improved understanding of brain metabolism at the cellular level and its roles in disease requires preventing metabolic disruption during assays and harvest of brain, brain slices, or cultured cells.

The extraordinary prevalence of metabolomic studies that use inappropriate assay procedures and/or harvest methods (see supplemental file 1 in reference Dienel and Nowak Jr 2025) indicates widespread lack of knowledge in the neuroscience and metabolomics communities of the importance and consequences of flawed methodology. For this reason, illustrative examples of metabolite assays and harvest procedures using inappropriate methods are evaluated in more detail below. A goal of this discussion is to provide non-specialist neuroscientists who do not have a strong background in metabolism with a perspective of key issues, sources of potential artifacts, and the importance of knowledge of the relevant literature, while also being informative to specialists.

### Isoflurane anesthesia

Isoflurane is a volatile anesthetic commonly used to prevent movement artifacts during in vivo ^1^H MRS metabolite assays, and it can be a useful tool for studies of brain metabolism and function (e.g Flatt et al. 2021, Wei et al. 2024), but it is not appropriate for comparisons of glycolytic metabolism, lactate accumulation, and levels of other metabolites in the metabolome (e.g Makaryus et al. 2011, Boretius et al. 2013, Dewan et al. 2020, Frame et al. 2024, Jimenez-Blasco et al. 2024). Isoflurane reduces the cerebral metabolic rate for glucose (CMR_glc_) by ~50% throughout rodent and human brain (Maekawa et al. 1986, Alkire et al. 1997, Lenz et al. 1998, Toyama et al. 2004), reduces CMRO_2_ in human brain (Madsen et al. 1987, Algotsson et al. 1988), affects many neurotransmitter systems in a dose-dependent manner (Müller et al. 2011, Speigel et al. 2023), causes rapid 3–4-fold increases in extracellular lactate (Horn and Klein 2010), and alters the extracellular (Baer et al. 2020) and tissue metabolomes (Makaryus et al. 2011, Boretius et al. 2013). Isoflurane rapidly and reversibly elevates brain lactate level by ~6-fold, with smaller percentage increases in levels of alanine, N-acetylaspartate (NAA), total creatine, choline-containing compounds, myo-inositol, GABA, and glutamate, a small decrease in glutamine, and nearly 50% fall in glucose level; these changes were ascribed to stimulation of adrenergic pathways in conjunction with inhibition of the respiratory chain (Boretius et al. 2013). In fact, inhibition of the mitochondrial electron transport chain complex I, the entry point for NADH oxidation, is proposed to be a primary mechanism of action of isoflurane (Jung et al. 2022). Effects of isoflurane on metabolite levels in MRS assays can be minimized by discontinuing its use in conjunction with a muscle relaxant to reduce movement artifacts (Boretius et al. 2013). Recently-developed procedures for MRS assays in awake mice (Laxer et al. 2025, Molhemi et al. 2025, Ono et al. 2025) eliminate complications of anesthesia. To sum up, it well known that MRS-measurable components of the metabolome and glucose metabolic pathway fluxes in extracts and in living brain are distorted by isoflurane (e.g Molhemi et al. 2025, Ono et al. 2025).

When isoflurane is used during tissue harvest by decapitation (e.g Dewan et al. 2020) or during brain perfusion followed by decapitation (e.g Koronowski et al. 2018, Loppi et al. 2023) it artifactually alters the metabolome determined in brain extracts by superimposing perimortem disruption of glucose metabolism on postmortem ischemia. Glycolytic metabolite studies in mice by Jimenez-Blasco et al (Jimenez-Blasco et al. 2024). used in vivo ^1^H MRS assays under isoflurane anesthesia. The significance of the ~55% higher concentration of lactate in the experimental versus control brain, along with small decreases in reduced glutathione and NAA plus N-acetylaspartylglutamate (Jimenez-Blasco et al. 2024), is questioned because isoflurane alters lactate and NAA levels (Boretius et al. 2013), and the separate effects of isoflurane and genotype on MRS-measurable metabolites were not established.

Anesthetics have different effects on metabolism that vary with class, dose, and duration, so the examples provided for isoflurane do not apply to other types of anesthetics (e.g Makaryus et al. 2011), although other halogenated volatile anesthetics may have similar effects (e.g Boretius et al. 2013). Nevertheless, it is best to avoid use of anesthetics for metabolic studies and euthanasia/tissue harvest, but, if required, the results must be interpreted with caution and include appropriate controls.

### CO_2_ asphyxiation

CO_2_ asphyxiation is a common euthanasia method that is not suitable for metabolic studies because it causes hypercarbia, tissue acidification, and hypoxia-anoxia, followed by postmortem ischemia after decapitation. CO_2_ asphyxiation drastically changed the metabolome and lipidome (Trépanier et al. 2017, Hennebelle et al. 2019, Sylvestre et al. 2022) and levels of many neurotransmitters and their metabolites (Wasek et al. 2018). Exposure to 30% CO_2_ for 5 min caused intracellular acidosis (CO_2_ + H_2_O ↔ H_2_CO_3_ ↔ H^+^ + HCO_3_^−^) that probably inhibited PFK activity (PFK has a steep pH-activity profile Trivedi and Danforth 1966, Halperin et al. 1969), increased glucose and glucose-6-phosphate levels (2.7- and 1.6-fold, respectively), and depressed CMR_glc_ by 43%, while increasing blood flow and CMRO_2_; downstream glycolytic metabolites, TCA cycle intermediates, and glutamate were consumed as alternative substrates in place of glucose (Folbergrova et al. 1972, Folbergrova et al. 1974, Folbergrova et al. 1975, Miller et al. 1975). For example, 30% CO_2_ for 5 min reduced levels of pyruvate by 65%, lactate by 70%, α-glycerophosphate by 45%, citrate by ~40%, α-ketoglutarate by ~70%, and malate by ~60% (Miller et al. 1975). Five min exposure to 30–45% CO_2_ decreased glutamate by 25% and increased aspartate levels by 50% (Folbergrova et al. 1975, Miller et al. 1975), whereas levels of both amino acids were elevated after 2 min of unspecified CO_2_ level (Sylvestre et al. 2022). The magnitude and duration of CO_2_ exposure govern its metabolic effects.

A likely scenario during ~5 min CO_2_ asphyxiation-euthanasia in any study that used this harvest procedure is that unlabeled and ^13^C-labeled glucose from blood may accumulate in brain by 2.5–3-fold, with simultaneous consumption of downstream lactate and glycolytic and TCA cycle intermediates to reduce the in vivo levels and labeling of these compounds. Concurrent oxidation of glutamate would bring its labeled and unlabeled carbon skeletons from the large glutamate pool into the TCA cycle, artifactually altering the concentrations of cycle intermediates and [^13^C]glucose labeling fractions. Then, postmortem ischemia will cause rapid consumption of unlabeled glucose and glycogen and [^13^C]glucose, accumulation of lactate, distortion of levels and labeling of glycolytic and TCA cycle intermediates, and depletion of high-energy compounds. Energy failure during harvest will also disrupt metabolism of lipids, lipid signaling compounds, neurotransmitters and their metabolites, and other labile compounds in many pathways.

Dobrowolski et al (Dobrowolski et al. 2022). harvested liver and brain from phenylketonuria-model and wildtype mice for evaluation of effects of chronic hyperphenylalanemia on the metabolome. The animals were euthanized with CO_2_ but no other harvest details were provided. The perimortem and postmortem conditions will substantially alter identified changes in glycolytic, lipid, redox, and other metabolites. The effects of genotype, hypercarbia, and anoxia/ischemia were not resolved.

Glucose hypometabolism is a common feature of AD (Gibson and Shi 2010), and metabolic studies in AD mice are used to understand the basis for metabolic deficits in AD. Knörnschild et al (Knörnschild et al. 2025) used CO_2_ for euthanasia of wildtype and AD model mice, followed by dissection and freezing of hippocampus and cerebral cortex, with the entire procedure taking 5–10 min. Ex-vivo high-resolution magic angle spinning ^1^H NMR measurements characterized the metabolomes by genotype and brain region, and identified changes by means of volcano plots and principal component analyses. However, the data analysis did not account for the consequences of CO_2_ asphyxiation and postmortem ischemia on glucose metabolism, oxidative stress, and other pathways.

Minhas et al (Minhas et al. 2024) studied glucose metabolism in AD model mice euthanized by CO_2_ asphyxiation, cervical dislocation, and decapitation, followed by hippocampal dissection and ‘flash’ freezing. The experimental paradigm was more complex than the above two studies because assays of unlabeled metabolite levels and metabolomic profiles were supplemented with [U-^13^C]glucose isotope tracing and matrix-assisted laser desorption ionization mass-spectrometry (MALDI) metabolite images, and is therefore described in more detail. In addition, parallel [^13^C]glucose isotope tracing and metabolic studies were carried out in cultured cells and hippocampal slices (Minhas et al. 2024). The in vitro data were also likely to be influenced by perimortem ischemia because the cultured cells used for assays of hexose phosphates and glycolytic intermediates were washed with a 5% mannitol aqueous solution at room temperature, then immersed in methanol. Human-derived cultured neurons and astrocytes were washed, pelleted, then frozen for metabolomic analysis. The wash durations and any metabolite washout were not stated, nor was slice harvest described.

During harvest-evoked ischemia, glycolytic flux will be upregulated to consume labeled and unlabeled glucose and unlabeled glycogen in vivo and in vitro, artifactually changing the concentrations and fractional ^13^C-labeling of glycolytic and TCA cycle metabolites. The concentration ranges of glycolytic and TCA cycle intermediates are very low (~0.01–0.5 μmol/g) compared with glucose and lactate (~1–2.5 and 0.6–1.5 μmol/g, respectively), and glutamate and aspartate levels (~12 and 3 μmol/g respectively) (Lowry et al. 1964, Goldberg et al. 1966, Veech et al. 1973, Miller et al. 1975, Srivastava et al. 2012). Rapid pool turnover (concentration divided by flux) facilitates changes in concentration and fractional labeling during ischemia. For example, the range of turnover times for the glycolytic and TCA cycle intermediates in mouse brain with a whole brain mean glucose utilization rate of 0.5 μmol/g/min (Qin and Smith 2007) (i.e. triose flux = 1 μmol/g/min) is ~0.01–0.5 min or 0.6–30s. Detailed time courses of ischemic changes in levels and pathway rates for most other labile metabolites have not, to my knowledge, been established, but they are likely rapid, extensive, and time dependent.

Thus, identified markers for abnormal glucose metabolism in the AD mice in vivo and in vitro were artifactually affected by the CO_2_/decapitation, wash, and harvest procedures. These markers include glucose, pyruvate, lactate, citrate, and malate, at least half of the 13 significantly-altered metabolites common to all AD models and about half of the identified compounds in the volcano plot and heat maps; in addition, the ^13^C-labeling patterns and MALDI images would be affected by unknown magnitudes (Figs. 2–6, S7, S8 in reference Minhas et al. 2024). Reporting metabolite levels as fold-changes and isotope tracing as fractions labeled (Minhas et al. 2024) prevents data evaluation and literature comparisons to known concentrations and pathway rates. Interpretive uncertainties arise because the analysis did not distinguish between effects of genotype, treatment paradigm, or peri- and postmortem conditions. The major conclusions need to be reconsidered.

## Concluding comments

Serious concerns arising from three major issues, effects of anesthesia on metabolism during assays and harvest, perimortem conditions that alter metabolism and metabolite levels, and procedures that do not stop enzyme activity at harvest, require reevaluation of roles of metabolism in normal brain and in animal models for neurological diseases. Studies described above are instructive because they reveal a variety of sources for artifactual metabolite and metabolomic data, isotope tracing profiles, and metabolite spatial images that may not have been identified without detailed evaluation and comparison with the relevant literature. Most past attention has been placed brain sampling procedures, with little attention to fixation of tissue slices or cultured cells. Cultured cells are commonly used to confirm and extend in vivo results in models for disease, and future studies need to quantitatively evaluate wash and harvest methods.

Metabolic studies using appropriate assay and harvest methods with reporting of absolute concentrations and pathway rates minimize artifacts and interpretive uncertainties. Metabolomics studies use advanced technologies to separate and quantify tens-to-thousands of metabolites and spatially-resolve brain metabolite distributions. Accurate characterization of a metabolome requires high-quality samples in which enzymatic activities are quenched at harvest. When this long-established knowledge base is not applied to metabolomics studies, investigator time, valuable research grant funds, animal use, and technical resources are misapplied. Anesthesia, perimortem conditions, and tissue harvest in vivo and in vitro are critical elements of metabolomics studies that must be more-widely recognized and implemented by researchers, funding agencies, editors, and reviewers.

## Supplementary Material

supplementary_file_for_export_kvag011

## Data Availability

Data supporting the findings of this study are available in the cited references and can be accessed from those publications.
