# Supplementary material for: Anesthesia, perimortem conditions, and brain harvest method govern the measured metabolome: analysis of substandard samples generates flawed metabolite levels
Source: Oxf Open Neurosci. 2026 Aug 26;5:kvag011. doi: 10.1093/oons/kvag011 (PMC13550777; doi:10.1093/oons/kvag011)
Supplement: Supplementary file 1 [file supplementary_file_for_export_kvag011.docx]

**22-Jun-2026**

Dear Dr. Dienel,

Manuscript ID OXFNSC-2026-018 entitled "Brain harvest method governs the measured metabolome: Analysis of substandard samples generates flawed metabolite levels" which you submitted to the Oxford Open Neuroscience, has been reviewed.  The comments of the reviewer(s) are included at the bottom of this letter.

The reviewer(s) have recommended publication, but also suggest some minor revisions to your manuscript.  Therefore, I invite you to respond to the reviewer(s)' comments and revise your manuscript within the next 30 days of todays date.

Please note that this journal operates with transparent peer review. This means that if your submission is accepted for publication, the full peer review history of your article will publish online alongside your article. This includes reviewer comments, editor decision letters, and your author responses.

To revise your manuscript, log into https://mc.manuscriptcentral.com/oxfnsc and enter your Author Centre, where you will find your manuscript title listed under "Manuscripts with Decisions."  Under "Actions," click on "Create a Revision."  Your manuscript number has been appended to denote a revision.

You may also click the below link to start the revision process (or continue the process if you have already started your revision) for your manuscript. If you use the below link you will not be required to login to ScholarOne Manuscripts.

*** PLEASE NOTE: This is a two-step process. After clicking on the link, you will be directed to a webpage to confirm. ***

https://mc.manuscriptcentral.com/oxfnsc?URL_MASK=e45078804df84eee8dc7926cb6133dd8


You will be unable to make your revisions on the originally submitted version of the manuscript.  Instead, revise your manuscript using a word processing program and save it on your computer.  Please also highlight the changes to your manuscript within the document by using the track changes mode in MS Word or by using bold or colored text.

<strong>REMINDER:</strong> if you have not already done so, when submitting your revised manuscript, please include a short alternative-text description under the figure legend for each figure. Alt-text is required to ensure the accessibility of our publications. Guidance is available on the journal’s Author Guidelines webpage and at <a href="https://academic.oup.com/pages/for-authors/journals/preparing-and-submitting-your-manuscript" target="_new">https://academic.oup.com/pages/for-authors/journals/preparing-and-submitting-your-manuscript</a>

Once the revised manuscript is prepared, you can upload it and submit it through your Author Centre.

When submitting your revised manuscript, you will be able to respond to the comments made by the reviewer(s) in the space provided.  You can use this space to document any changes you make to the original manuscript.  In order to expedite the processing of the revised manuscript, please be as specific as possible in your response to the reviewer(s).

IMPORTANT:  Your original files are available to you when you upload your revised manuscript.  Please delete any redundant files before completing the submission.

Because we are trying to facilitate timely publication of manuscripts submitted to the Oxford Open Neuroscience, your revised manuscript should be uploaded within the next 30 days from todays date.  If it is not possible for you to submit your revision in a reasonable amount of time, we may have to consider your paper as a new submission.

Once again, thank you for submitting your manuscript to the Oxford Open Neuroscience and I look forward to receiving your revision.

Sincerely,
Dr. Orly Reiner
Senior Editor, Oxford Open Neuroscience
orly.reiner@weizmann.ac.il, orly.reiner@weizmann.ac.il


Reviewer: 1

Comments to the Author
This is a timely and important contribution.  As nicely summarized here, the issue of post-mortem changes in metabolites has been recognized for many It's, but has been ignored in a large number of recent metabolomic publications.  This is a very significant problem for the field, and the manuscript submitted here addresses it in a clear manner. A limitation though is that this issue was very thoroughly addressed by Dienel and Nowak (reference 2 in this manuscript, and it is unclear how the current submission advances this.

Suggestions for revision
1. The fixation issue extends to all tissues, not just brain, and perhaps this could be emphasized.

2. The sentence beginning on line 9 of page 5 is unclear – may need to be fleshed out a bit more.

3. Consider omitting the “INTERPRETIVE CONCERNS” section, or greatly abbreviating it.  If the manuscript ended at this point would stand on its own, despite its brevity.  The problem here is that the “INTERPRETIVE CONCERNS” section is actually a very detailed critique of two specific papers. There is no explanation as to why these two were singled out.  Many of the points made in the critique of these papers are outside the scope of the fixation issues, and this could unfortunately give the impression that it article is just a veiled “hit piece “on these articles rather than a more general call for awareness of the tissue fixation issue.

4. Alternatively, these two articles could be introduced as two illustrative examples among many in which fixation issues have likely contributed to artifactual results. In that case, it may be preferable to restrict the comments here specifically the in vivo studies in which this is most evident.


Reviewer: 2

Comments to the Author
This manuscript describes the changes that may occur subsequent to death due to failure to rapidly stop enzymatic activity and highlights some findings in the literature that may have arisen due to this failure. The author is drawing attention to a common issue in brain metabolic studies which is generally under appreciated by the community, whether knowingly or unknowingly, mostly for logistic reasons.
After summarising the changes that may arise due to post-mortem metabolism and highlighting the need to know how these changes may differ between pathology and control, the author gives some examples of where a combination of post-mortem changes along with reporting of metabolic ratios rather than absolute values may have led to insecure conclusions.
The work is well written and researched and should serve as a useful reference, along with the references contained therein.

Associate Editor
Comments to the Author:
Dear Author, after receiving the reviewer's comments, I will be pleased to receive your corrected version for consideration for publication. You work is timely and of great importance to improve preclinical research in brain metabolism. Overall, the greatest amendment is to diminished the tone from first person to a generic format. Below you will find each specific revision.

Reviewer 1:
This is a timely and important contribution. As nicely summarized here, the issue of post-mortem changes in metabolites has been recognized for many It's, but has been ignored in a large number of recent metabolomic publications. This is a very significant problem for the field, and the manuscript submitted here addresses it in a clear manner. A limitation though is that this issue was very thoroughly addressed by Dienel and Nowak (reference 2 in this manuscript, and it is unclear how the current submission advances this.

Suggestions for revision
1. The fixation issue extends to all tissues, not just brain, and perhaps this could be emphasized.

2. The sentence beginning on line 9 of page 5 is unclear – may need to be fleshed out a bit more.

3. Consider omitting the “INTERPRETIVE CONCERNS” section, or greatly abbreviating it. If the manuscript ended at this point would stand on its own, despite its brevity. The problem here is that the “INTERPRETIVE CONCERNS” section is actually a very detailed critique of two specific papers. There is no explanation as to why these two were singled out. Many of the points made in the critique of these papers are outside the scope of the fixation issues, and this could unfortunately give the impression that it article is just a veiled “hit piece “on these articles rather than a more general call for awareness of the tissue fixation issue.

4. Alternatively, these two articles could be introduced as two illustrative examples among many in which fixation issues have likely contributed to artifactual results. In that case, it may be preferable to restrict the comments here specifically the in vivo studies in which this is most evident.

Reviewer 2:
This manuscript describes the changes that may occur subsequent to death due to failure to rapidly stop enzymatic activity and highlights some findings in the literature that may have arisen due to this failure. The author is drawing attention to a common issue in brain metabolic studies which is generally under appreciated by the community, whether knowingly or unknowingly, mostly for logistic reasons.
After summarising the changes that may arise due to post-mortem metabolism and highlighting the need to know how these changes may differ between pathology and control, the author gives some examples of where a combination of post-mortem changes along with reporting of metabolic ratios rather than absolute values may have led to insecure conclusions.
The work is well written and researched and should serve as a useful reference, along with the references contained therein.

**Author's Response to Decision Letter for (OXFNSC-2026-018)**

Associate Editor
Comments to the Author:
Dear Author, after receiving the reviewer's comments, I will be pleased to receive your corrected version for consideration for publication. You work is timely and of great importance to improve preclinical research in brain metabolism. Overall, the greatest amendment is to diminished the tone from first person to a generic format. Below you will find each specific revision.

Response: Thank you for the positive comments. All comments and suggestions by the Editor and Reviewers were addressed and identified by ‘track change’ in the manuscript. A clean copy of the main text was also uploaded. The first person format was changed for all instances noted after the comments by Reviewer 2, below.


Reviewer 1:
This is a timely and important contribution. As nicely summarized here, the issue of post-mortem changes in metabolites has been recognized for many It's, but has been ignored in a large number of recent metabolomic publications. This is a very significant problem for the field, and the manuscript submitted here addresses it in a clear manner. A limitation though is that this issue was very thoroughly addressed by Dienel and Nowak (reference 2 in this manuscript, and it is unclear how the current submission advances this.

Response: Thank you for your positive comments.

Given the broad lack of knowledge in the neuroscience and metabolomics fields of the effects of inappropriate conduct of metabolic assays and tissue harvest, discussion of the key issues in different venues is important. The current manuscript is for a special issue entitled, “Brain metabolism across disorders: Linking molecular mechanisms to clinical interventions”, and a commentary addressing the critical importance of assay procedures, perimortem conditions, and brain harvest method for metabolic studies provides an important perspective for authors and readers of this special issue.

Suggestions for revision
1. The fixation issue extends to all tissues, not just brain, and perhaps this could be emphasized.

Response: Thank you for emphasizing this important point. Edits were made to the abstract to change ‘brain’ to ‘tissue’, a sentence was added to the Introduction, and a brief paragraph describing the use of freeze-clamping to harvest other organ tissues was added to the end of the section ‘Enzyme Inactivation’.

2. The sentence beginning on line 9 of page 5 is unclear – may need to be fleshed out a bit more.

Response: The sentence was revised to improve clarity.

3. Consider omitting the “INTERPRETIVE CONCERNS” section, or greatly abbreviating it. If the manuscript ended at this point would stand on its own, despite its brevity. The problem here is that the “INTERPRETIVE CONCERNS” section is actually a very detailed critique of two specific papers. There is no explanation as to why these two were singled out. Many of the points made in the critique of these papers are outside the scope of the fixation issues, and this could unfortunately give the impression that it article is just a veiled “hit piece “on these articles rather than a more general call for awareness of the tissue fixation issue.

Response: This is an important point, and the revised manuscript presents the material more clearly with respect to three critical aspects of metabolite/metabolomic studies that are separate but interrelated issues: anesthesia, perimortem conditions, and tissue harvest.

To broaden the scope of the commentary and improve clarity, the title of the manuscript was changed to “Anesthesia, perimortem conditions, and brain harvest method govern the measured metabolome: Analysis of substandard samples generates flawed metabolite levels”.

The abstract and text were revised, and the text includes a brief discussion of use of anesthesia (with a focus on isoflurane) during in vivo MRS assays to prevent motion artifacts, the use of anesthesia during tissue harvest, the impact of perimortem conditions (e.g., perfusion of the brain to remove blood and CO2 exposure during euthanasia), and the brain harvest method to prevent postmortem ischemia.

The two papers used as illustrative examples focus on timely important issues in brain metabolism and neurological disease. One study used isoflurane anesthesia during in vivo metabolite assays designed to evaluate glycolysis, but isoflurane interferes with oxidative metabolism and increases lactate levels, confounding data interpretation. That study did not specify the harvest method, but probably used decapitation, subjecting the tissue to postmortem ischemia, causing artifacts in the metabolome, volcano plot, and heat map. The other study harvested the brain after CO2 asphyxiation that would markedly distort the metabolome and [13C]glucose labeling. Both studies used cultured cells to extend and complement the in vivo studies, but aspects of these comparisons between immature cultured cells and adult brain appear to be discordant. The impact of the experimental procedures in these studies applies to many other studies, some of which are also cited.

The discussion was detailed because many neuroscientists do not have expertise in metabolism, and critical analysis of any report requires that the details be presented so that general readers and knowledgeable readers can understand and evaluate the basis for criticism, then draw their own conclusions.


4. Alternatively, these two articles could be introduced as two illustrative examples among many in which fixation issues have likely contributed to artifactual results. In that case, it may be preferable to restrict the comments here specifically the in vivo studies in which this is most evident.

Response: These two papers were chosen as illustrative examples of the impact of anesthesia, perimortem conditions, and tissue harvest procedure, with emphasis on the in vivo studies. The reasoning for their inclusion is explained. Discussion of in vitro studies that were intended to support and extend the in vivo studies was retained but shortened somewhat.


Reviewer 2:
This manuscript describes the changes that may occur subsequent to death due to failure to rapidly stop enzymatic activity and highlights some findings in the literature that may have arisen due to this failure. The author is drawing attention to a common issue in brain metabolic studies which is generally under appreciated by the community, whether knowingly or unknowingly, mostly for logistic reasons.
After summarising the changes that may arise due to post-mortem metabolism and highlighting the need to know how these changes may differ between pathology and control, the author gives some examples of where a combination of post-mortem changes along with reporting of metabolic ratios rather than absolute values may have led to insecure conclusions.
The work is well written and researched and should serve as a useful reference, along with the references contained therein.

Response: Thank you for your positive comments.

**07-Jul-2026**

Dear Dr. Dienel,

Manuscript ID OXFNSC-2026-018.R1 entitled "Anesthesia, perimortem conditions, and brain harvest method govern the measured metabolome: Analysis of substandard samples generates flawed metabolite levels" which you submitted to the Oxford Open Neuroscience, has been reviewed.  The comments of the reviewer(s) are included at the bottom of this letter.

The reviewer(s) have recommended publication, but also suggest some major revisions to your manuscript.  Therefore, I invite you to respond to the reviewer(s)' comments and revise your manuscript within the next 60 days from todays date.

Please note that this journal operates with transparent peer review. This means that if your submission is accepted for publication, the full peer review history of your article will publish online alongside your article. This includes reviewer comments, editor decision letters, and your author responses.

To revise your manuscript, log into https://mc.manuscriptcentral.com/oxfnsc and enter your Author Centre, where you will find your manuscript title listed under "Manuscripts with Decisions."  Under "Actions," click on "Create a Revision."  Your manuscript number has been appended to denote a revision.

You may also click the below link to start the revision process (or continue the process if you have already started your revision) for your manuscript. If you use the below link you will not be required to login to ScholarOne Manuscripts.

*** PLEASE NOTE: This is a two-step process. After clicking on the link, you will be directed to a webpage to confirm. ***

https://mc.manuscriptcentral.com/oxfnsc?URL_MASK=56a73441b188485ea53f87b0a439c477

You will be unable to make your revisions on the originally submitted version of the manuscript.  Instead, revise your manuscript using a word processing program and save it on your computer.  Please also highlight the changes to your manuscript within the document by using the track changes mode in MS Word or by using bold or colored text.

<strong>REMINDER:</strong> if you have not already done so, when submitting your revised manuscript, please include a short alternative-text description under the figure legend for each figure. Alt-text is required to ensure the accessibility of our publications. Guidance is available on the journal’s Author Guidelines webpage and at <a href="https://academic.oup.com/pages/for-authors/journals/preparing-and-submitting-your-manuscript" target="_new">https://academic.oup.com/pages/for-authors/journals/preparing-and-submitting-your-manuscript</a>

Once the revised manuscript is prepared, you can upload it and submit it through your Author Centre.

When submitting your revised manuscript, you will be able to respond to the comments made by the reviewer(s) in the space provided.  You can use this space to document any changes you make to the original manuscript.  In order to expedite the processing of the revised manuscript, please be as specific as possible in your response to the reviewer(s).

IMPORTANT:  Your original files are available to you when you upload your revised manuscript.  Please delete any redundant files before completing the submission.

Because we are trying to facilitate timely publication of manuscripts submitted to the Oxford Open Neuroscience, your revised manuscript should be uploaded within the next 60 days from todays date.  If it is not possible for you to submit your revision in a reasonable amount of time, we may have to consider your paper as a new submission.

Once again, thank you for submitting your manuscript to the Oxford Open Neuroscience and I look forward to receiving your revision.

Sincerely,
Dr. Orly Reiner
Senior Editor, Oxford Open Neuroscience
orly.reiner@weizmann.ac.il, orly.reiner@weizmann.ac.il


Reviewer: 2

Comments to the Author
The author has addressed the issues raised.

Reviewer: 1

There is a need to properly address the following comments: 
3. Consider omitting the “INTERPRETIVE CONCERNS” section, or greatly abbreviating it. If the manuscript ended at this point would stand on its own, despite its brevity. The problem here is that the “INTERPRETIVE CONCERNS” section is actually a very detailed critique of two specific papers. There is no explanation as to why these two were singled out. Many of the points made in the critique of these papers are outside the scope of the fixation issues, and this could unfortunately give the impression that it article is just a veiled “hit piece “on these articles rather than a more general call for awareness of the tissue fixation issue.

4. Alternatively, these two articles could be introduced as two illustrative examples among many in which fixation issues have likely contributed to artifactual results. In that case, it may be preferable to restrict the comments here specifically the in vivo studies in which this is most evident.

Associate Editor
Comments to the Author:
Dear Dr. Dienel, many thanks for taking the reviewer's comments into consideration, and for apply them to the manuscript. This Perspective is timely and I am positive that it will help advance preclinical research in brain metabolism.

**Author’s response to reviewer/editor comments on Manuscript ID OXFNSC-2026-018.**R1 entitled "Anesthesia, perimortem conditions, and brain harvest method govern the measured metabolome: Analysis of substandard samples generates flawed metabolite levels"


Reviewer: 2

Comments to the Author
The author has addressed the issues raised.

Reviewer: 1

There is a need to properly address the following comments:
3. Consider omitting the “INTERPRETIVE CONCERNS” section, or greatly abbreviating it. If the manuscript ended at this point would stand on its own, despite its brevity. The problem here is that the “INTERPRETIVE CONCERNS” section is actually a very detailed critique of two specific papers. There is no explanation as to why these two were singled out. Many of the points made in the critique of these papers are outside the scope of the fixation issues, and this could unfortunately give the impression that it article is just a veiled “hit piece “on these articles rather than a more general call for awareness of the tissue fixation issue.

4. Alternatively, these two articles could be introduced as two illustrative examples among many in which fixation issues have likely contributed to artifactual results. In that case, it may be preferable to restrict the comments here specifically the in vivo studies in which this is most evident.

Associate Editor
Comments to the Author:
Dear Dr. Dienel, many thanks for taking the reviewer's comments into consideration, and for apply them to the manuscript. This Perspective is timely and I am positive that it will help advance preclinical research in brain metabolism.


Response: The manuscript was extensively revised, as described in the marked copy of the R2 version of the manuscript.

**06-Aug-2026**

Dear Dr. Dienel,

It is a pleasure to accept your revised manuscript entitled "Anesthesia, perimortem conditions, and brain harvest method govern the measured metabolome: Analysis of substandard samples generates flawed metabolite levels" in its current form for publication in the Oxford Open Neuroscience.  The comments of the reviewer(s) who reviewed your manuscript are included at the foot of this letter.

Please note that this journal operates with transparent peer review. This means that  the full peer review history of your article will publish online alongside your article. This includes reviewer comments, editor decision letters, and your author responses.

Next steps
You will receive an email from no-reply@scipris.com within roughly one week. This is your invitation to sign up for an account with SciPris, Oxford University Press’ author portal hosted by Aptara. You will need to create an account if you do not already hold one. Please register or log into your account and follow the online instructions which will guide you through signing your licence and paying the APC. The email and the portal have clearly signposted support options if you need any help during this process.

Please note that SciPris is a completely different system from ScholarOne, so your credentials to submit your manuscript here will not work there. Once you’ve created a SciPris account, you will be able to use it whenever you publish with Oxford Open Neuroscience or any OUP journal. Please note that OUP will only ever request payment for applicable fees be made via SciPris or to an OUP bank account. If you ever have concerns about the legitimacy of a request, please do not hesitate to contact a customer services agent via the SciPris portal or directly via oupsupport@scipris.com.

Thank you for your fine contribution.  On behalf of the Editors of the Oxford Open Neuroscience, we look forward to your continued contributions to the Journal.

Sincerely,
Dr. Orly Reiner
Senior Editor, Oxford Open Neuroscience
orly.reiner@weizmann.ac.il, orly.reiner@weizmann.ac.il


Reviewer: 3

Comments to the Author
Dear author,
I would like to congratulate you on the quality of the document. The substantial revision made to this version address the original comments, and excel the quality and readibility of an important issue in preclinical research that over the years has set to a side.

This manuscript tackles weaknesses at in vivo and in vitro level and I am confident that the piece will help advance the field of brain metabolism.

Best,

Reviewer: 1

Comments to the Author
I have no further comments or concerns.  Thank you for this important contribution.

Associate Editor
Comments to the Author:
Dear Dr. Deimel,
Thank you so much for the corrections made to the document. I beleive the content of your manuscript is of high relevance to the field and will serve as a template to improve sample acquisition and interpretation.
Best,
